# Co-creation and User Perspectives for Upper Limb Prosthetics

**DOI:** 10.3389/fnbot.2021.689717

**Published:** 2021-07-09

**Authors:** Hannah Jones, Sigrid Dupan, Matthew Dyson, Agamemnon Krasoulis, Laurence P. J. Kenney, Margaret Donovan-Hall, Kaveh Memarzadeh, Sarah Day, Maxford Coutinho, Kianoush Nazarpour

**Affiliations:** ^1^Edinburgh Neuroprosthetics Laboratory, School of Informatics, University of Edinburgh, Edinburgh, United Kingdom; ^2^Intelligent Sensing Laboratory, School of Engineering, Newcastle University, Newcastle upon Tyne, United Kingdom; ^3^School of Health and Society, University of Salford, Manchester, United Kingdom; ^4^School of Health Sciences, University of Southampton, Southampton, United Kingdom; ^5^Orthopaedic Research UK, London, United Kingdom; ^6^Department of Biomedical Engineering, University of Strathclyde, Glasgow, United Kingdom; ^7^Department of Plastic Surgery, Royal Victoria Infirmary, Newcastle upon Tyne, United Kingdom

**Keywords:** co-creation, collaboration, stakeholders, upper limb prosthetics, user-centred approach

## Abstract

People who either use an upper limb prosthesis and/or have used services provided by a prosthetic rehabilitation centre, experience limitations of currently available prosthetic devices. Collaboration between academia and a broad range of stakeholders, can lead to the development of solutions that address peoples' needs. By doing so, the rate of prosthetic device abandonment can decrease. Co-creation is an approach that can enable collaboration of this nature to occur throughout the research process. We present findings of a co-creation project that gained user perspectives from a user survey, and a subsequent workshop involving: people who use an upper limb prosthesis and/or have experienced care services (users), academics, industry experts, charity executives, and clinicians. The survey invited users to prioritise six themes, which academia, clinicians, and industry should focus on over the next decade. The prioritisation of the themes concluded in the following order, with the first as the most important: function, psychology, aesthetics, clinical service, collaboration, and media. Within five multi-stakeholder groups, the workshop participants discussed challenges and collaborative opportunities for each theme. Workshop groups prioritised the themes based on their discussions, to highlight opportunities for further development. Two groups chose function, one group chose clinical service, one group chose collaboration, and another group chose media. The identified opportunities are presented within the context of the prioritised themes, including the importance of transparent information flow between all stakeholders; user involvement throughout research studies; and routes to informing healthcare policy through collaboration. As the field of upper limb prosthetics moves toward in-home research, we present co-creation as an approach that can facilitate user involvement throughout the duration of such studies.

## Introduction

The aim of the paper is to highlight development areas for academia, healthcare sector, and industry to explore from a user-centred perspective. The presented findings from a user survey and multi-stakeholder workshop provide examples that could inform such development. In addition, the paper aims to present how co-creation can be used as an approach when researching with users. In this paper, with the term *users*, we refer to people who either use an upper limb prosthesis and/or have used services provided by a prosthetic rehabilitation centre. We do not make a distinction between acquired or congenital limb difference conditions, unless explicitly indicated.

People who use an upper limb prosthesis experience a range of challenges, such as functional limitations, and psychological support provision. Advanced prosthetic solutions, developed within academia, aim to meet the needs of prosthesis users (Nazarpour, 2020a). However, currently available prostheses fall short in addressing user needs, for example: function and sensory feedback, leading to device abandonment rates of up to 44% (Postema et al., 1999, 2016; Davidson, 2002; Biddiss and Chau, 2007a,b; Østlie et al., 2012; Sugawara et al., 2018; Salminger et al., 2020). For body powered and electronic devices, the abandonment rates of 26 and 23% have been reported (Biddiss and Chau, 2007b).

Upper limb prosthesis users exhibit a wide range of wear and use time (Chadwell et al., 2018). However, 74% of non-users would reconsider using a prosthesis, if technological improvements were made at a reasonable cost (Biddiss and Chau, 2007a). Biddiss et al. (Biddiss et al., 2007) state that efforts to address user design priorities are critical in addressing the needs of users who choose not to use a prosthesis.

In an attempt to improve user satisfaction, and ultimately limit device abandonment, studies have been conducted to identify user requirements (see Cordella et al., 2016; Kumar et al., 2019 for extended reviews). Reported priorities include comfort (Biddiss et al., 2007; Jang et al., 2011) appearance (Kyberd et al., 2007; Jang et al., 2011), weight and improved function (Biddiss et al., 2007; Kyberd et al., 2007; Østlie et al., 2012; Engdahl et al., 2015; Luchetti et al., 2015). However, Kumar et al. (2019) point out that literature shows contradictions, where devices that meet the identified requirements are not necessarily accepted. Therefore, the inclination to accept new technologies is not experienced by all. People within younger age groups, with acquired limb loss, and have a unilateral limb difference may be more interested in adopting new technologies (Engdahl et al., 2017).

Identified challenges are being addressed by academia (Cipriani et al., 2010; Wheeler et al., 2019). However, there is evidence that laboratory-based metrics and findings are not always consistent with clinical outcomes (Vujaklija et al., 2017). Academic research is transitioning toward a combination of laboratory-based studies, and testing devices and systems within people's home environment, to enable clinical translation (Cuberovic et al., 2019; Simon et al., 2019; Hahne et al., 2020; Schofield et al., 2020; Wu et al., 2021). For clinical and user acceptance to be achieved, academia could benefit from collaborating with a variety of stakeholders, such as users, healthcare providers, policy makers, industry specialists, and medical charities.

One approach to enable this form of collaboration is co-creation, which facilitates the development of knowledge between academics and multiple stakeholders (Cairney and Oliver, 2017; Hickey et al., 2018; Nazarpour, 2020b; Jones et al.). Co-creation can be used interchangeably with terminology, such as co-design, leading to multiple definitions (De Koning et al.). For clarity, we refer to co-creation as a broad approach that facilitates collaboration with multiple stakeholders throughout research studies (Pearce et al., 2020).

This paper presents findings from a user survey and a 1-day co-creation workshop, entitled: “The Future of Prosthetics: A User Perspective.” Multiple stakeholders attended the workshop: users, academics, clinicians, industry experts, and charity executives. The workshop facilitated collaborative discussions on a range of challenges and opportunities within six themes: function, psychology, aesthetics, clinical service, collaboration, and media.

Based on the workshop findings, a vision as to how research can be conducted with multiple stakeholders, including users, is documented. Co-creation is presented as an approach that can facilitate such a vision, with an aim to develop new knowledge for clinical implementation. Although the workshop had representation from the National Health Service (NHS) in United Kingdom, the context and dialogue that was shared can be applicable to other countries with similar prosthetic care models.

## Methods

The methods used to formulate, deliver, and analyse the workshop spanned over the course of two phases: (A) pre-workshop development, (B) workshop delivery and analysis. The research methods outlined in this paper were approved by the local ethics committee at Newcastle University (ref: 13737/2018).

A. Pre-workshop Development

A small working group, comprising a congenital user, an acquired user, and an academic in the field of prosthetic research, identified six themes that are associated with prosthesis user experience. These themes were:

1. **Function:** The experience of using a prosthetic device, including the practicalities of putting on a prosthesis, compensatory movement, comfort, and adaptability to user needs.
2. **Psychology:** The psychological experience of living with limb difference, including interactions with clinical teams and services, confidence amongst differing social circles, and the thought process of using a device or healthcare service.
3. **Aesthetics:** The external form of a prosthetic device. Including how a device visually fits within a work environment or social occasions, social assumptions of what a prosthesis should look like, and how attention is drawn to or withdrawn from a device.
4. **Clinical Service:** The experience of receiving a clinical service, such as provided by the NHS, including clinical assessment process, interactions with clinicians and/or prosthetists, the care pathway and signposting users to other services (such as user network groups).
5. **Collaboration:** Collaboration across disciplines, and balancing long- and short-term research projects to bridge the gap between academic research and the prosthetic solutions that the clinical sector can currently provide.
6. **Media:** The effect media, including social media, has on users and its influence on informing the general public, including expectation management, reports of long-term solutions, and influencing societal assumptions on what the clinical sector can offer.

A pre-workshop survey was conducted to collect data on the six themes. The survey specifically requested users to prioritise the themes; for academia, the NHS, and industry to focus on over the next decade. The conclusion of the survey informed the workshop delivery and analysis. Out of the 12 survey participants, 11 attended the workshop.

B. Workshop Delivery and Analysis

The workshop had 26 people in attendance, including: users and their relatives, healthcare clinicians, academic researchers, industry specialists, and charity executives. Table 1 reports the counts of the participants in each stakeholder group with respect to gender.

**Table 1:** Co-creation workshop participants and their affiliations.

	**Acquired**	**Congenital**	**Relative**	**Academia**	**Industry**	**Healthcare**	**Charity**
Male	3	2	1	4	3	2	1
Female	4	2	1	1	0	2	0
Total	7	4	2	5	3	4	1

The workshop was organised into five multi-stakeholder groups, with at least one user and one academic per group (Table 2). The distribution of users across groups was also informed by the cause of limb absence: acquired or congenital (Table 2). Each group was formed of people that had no known prior working or clinical interaction.

**Table 2:** Co-creation workshop groups.

**Group**	**Acquired**	**Congenital**	**Relative**	**Academia**	**Industry**	**Healthcare**	**Charity**
1	1	1	1	1	0	0	0
2	0	1	0	1	0	1	0
3	1	2	0	1	1	1	0
4	3	0	1	1	1	1	0
5	2	0	0	1	1	1	1

**Figure 1:**
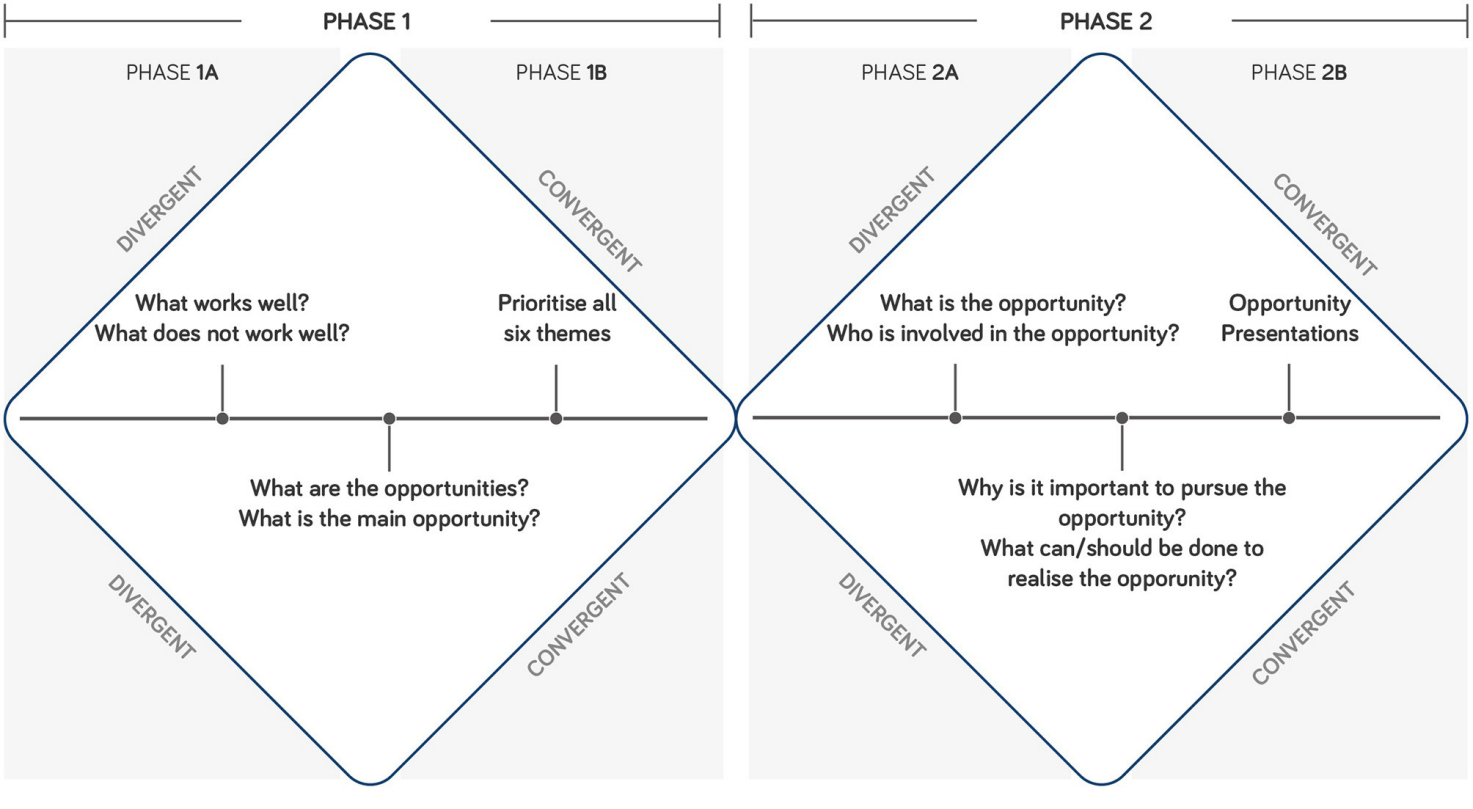
Workshop model, inspired by the Double Diamond Model by the Design Council (2019).

Figure 1 shows the workshop model, which was inspired by the Double Diamond model, developed by the Design Council (2019). This model combines divergent and convergent thinking that enabled each workshop group to: (1) identify opportunities across the six themes during Phase 1A; (2) prioritise the six themes in Phase 1B; and (3) explore ideas based on an identified opportunity from the top prioritised theme during Phase 2. The combination of divergent and convergent thinking provided a structure to the workshop and directed the questions; enabling groups to discuss themes, and form conclusions within a 1-day workshop.

### Workshop Model Phase 1

During Phase 1A, groups discussed challenges and opportunities for each theme. Structured worksheets were used to capture the conversations, encouraging participants to address what works well, what does not work well, what the opportunities are, and what the main opportunity is.

In the convergent section of Phase 1B, groups prioritised the six themes in order of importance to be addressed by academia, industry, NHS, and charity over the next 10 years.

### Workshop Model Phase 2

In Phase 2A, groups discussed a key opportunity from their top prioritised theme. Across the five groups: function (chosen by two groups), collaboration, clinical service, and media were explored as top prioritised themes within Phase 2A. Participants determined what the opportunity is; why it is important to pursue it; who should be involved; and what can/should be done to realise the opportunity. The workshop converged with each group presenting their chosen opportunity to all workshop attendees in Phase 2B.

Each group was assigned a facilitator to guide the themed sessions and capture data. The facilitator documented anonymised information on worksheets as hand-written data.

The data documented by the facilitators was analysed for each theme and cross referenced over all five groups. Patterns within the data were extracted and the commonalities across the groups were identified. The information presented in the Results section is exclusively based on the data that was documented on the worksheets by the facilitators during the workshop. GRIPP2, the Guidance for Reporting Involvement of Patients and Public (Staniszewska et al., 2017), was used as a tool during the write-up of this workshop. The tool highlights key aspects to document during studies that involve users, such as engagement methodology and results.

## Results

The results section is divided into pre-workshop survey and workshop content. Both sections present findings based on the six workshop themes. All opinions that are presented in italic quotation marks were sourced from workshop participants who are users, unless stated otherwise.

A. Pre-workshop Online Survey

**Figure 2:**
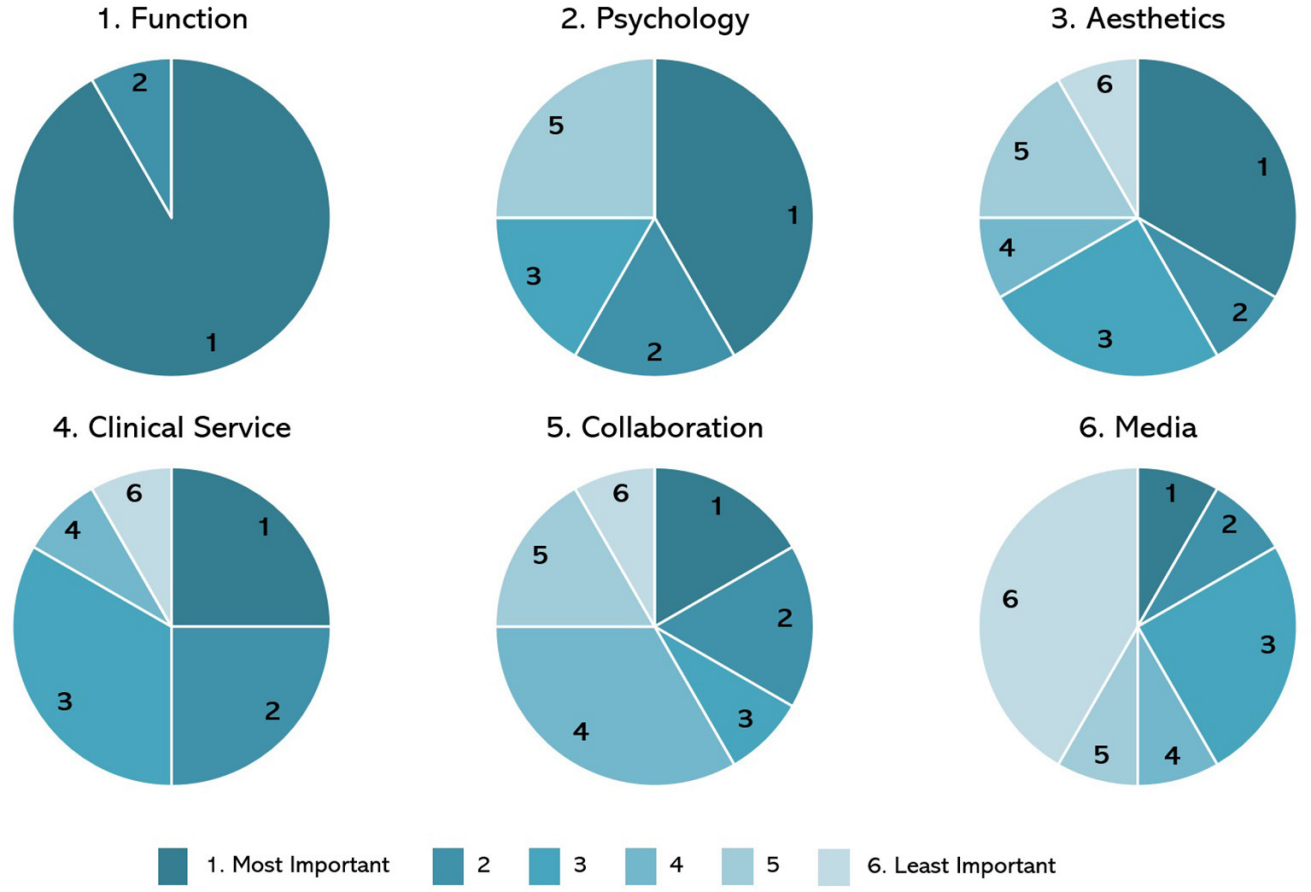
Pre-workshop survey results.

The order of importance sourced from the survey results is presented in Figure 2: function, psychology, aesthetics, clinical service, collaboration, and media. One user who took part in the survey, a minor, was assisted by their parent, and could not attend the workshop. The remaining 11 users attended the workshop, which happened after the survey data collection.

The results indicated that there was a variation in how each person prioritised the themes (Table 3). For example, seven users (A–G, Table 3) prioritised all six themes from one to six, whereas the remaining five users (H–L, Table 3) placed at least three themes as most important, with one respondent sharing that they thought “*all of these [themes] could be a 1 [most important]*.”

**Table 3:** Pre-workshop survey results per user.

**User**	**Function**	**Psychology**	**Aesthetics**	**Clinical Service**	**Collaboration**	**Media**
A	1	5	3	4	2	6
B	1	2	6	3	4	5
C	1	5	2	3	6	4
D	1	2	4	6	5	3
E	1	3	5	2	4	6
F	1	5	3	2	4	6
G	2	1	5	3	4	6
H	1	1	1	1	5	6
I	1	1	1	3	3	3
J	1	1	1	1	1	2
K	1	3	3	2	1	1
L	1	1	1	1	2	3

*Users A–G prioritised the themes from one to six. Users H–L placed at least three themes as most important. This distinction is indicated with two shades. Underlined user names represent those with congenital limb loss*.

The range of results indicate that to varying degrees, all themes were deemed important and therefore should be discussed equally throughout the workshop. One user shared the following comment: “*To be honest we find all of these subjects really important and should be addressed. We think function, how it looks and the experience you receive collecting your new limb are all as important as each other. But then, in order to move this technology along faster there must be collaboration and media interest*.”

B. Workshop Content

This section presents the data that was captured from all five groups for each of the six themes: function, psychology, aesthetics, clinical service, collaboration, and media. The themes are presented in order of perceived importance sourced from the pre-workshop survey results.

Opportunities are presented within the top prioritised themes (function, clinical service, collaboration, and media), as an outcome of Phase 1A (Figure 1). Each group selected their theme as the most important to be addressed by academia, industry, NHS, and charity over the next 10 years. The opportunities are initial ideas that highlight areas for further development within the field of upper limb prosthetics.

(1) *Function:* The functional performance of a prosthesis can be influenced by the expectation and the experience of a user. One user stated: “*Function is very individual and can be very client-centric*.” There was a resounding support for the view that user input to the prosthesis function development should be integrated into the design process.

Three groups discussed the physical weight of a prosthesis and how it can have varying issues. One participant, stated that a hand is “*heavy, but people can adapt*,” in comparison, an academic shared that “*[weight is a] huge issue*.”

The functionality of a prosthesis is limited to a number of tasks or grips. However, having a highly technological solution is not necessarily needed, with one user stating that “*sometimes the simple ones just work, and I don't need anything fancy*.” The specificity and requirements to conduct individual tasks were discussed by four groups. For one user, speed was not the key component of a highly functioning hand; rather a requirement to do specific tasks at specific times in specific scenarios.

Three groups discussed the topic of pursuing further research that is required to assess the value of prosthetics from a user perspective, by collecting live data instead of using asynchronous methods, such as retrospectively documenting experiences in a diary. There was also a suggestion to gather specific data on upper limb prosthetics, for example focusing solely on traumatic amputation, congenital limb difference, or illness related amputation. One of the three groups explored how this type of data could be used to inform future clinical provisions about the functionality of prosthetic hands. However, the same group stated that the culture of academic research tends to publish early-stage results without taking into account the variables that affect real-life use.

*Opportunities:* Groups 1 and 5 chose function as the most important theme at the end of Phase 1, to explore in Phase 2 (Figure 1). Group 1 focused their opportunity on a universal socket that enables different terminal devices, based on specific user needs. Group 5 explored the potential benefits in pursuing long-term studies to gain evidence of prosthetic devices that improve the quality of life, with an aim to drive the UK health system, toward funding multifunctional arms.

(2) *Psychology:* Psychological experiences are individual and have an association with the time one realises their limb absence: “[there is a] difference between amputees vs. congenital or if someone requested an amputation.”

Four groups discussed the psychological impact within social occasions, from both a negative and a positive viewpoint. For example, one user shared that “*interest in the artificial limb itself can be a boost, [as] focus goes from limb difference to technology*.” In contrast, a user shared that “*you get judged/people stare*,” and another remarked that “*getting a hand does not equal that you are OK*.”

The provision of psychological support is varied, mainly due to the governmental funding available to the local prosthetics service. There was a consensus that a stronger link should be created between psychological and clinical services in the context of limb loss, both in a general healthcare related context, and in providing specialised support. A group proposed that case workers would ideally work one-to-one with service users, but also appropriately involve close family and friends in the process, as this support network is important for psychological rehabilitation. To quantify the benefit of psychological support and justify interventions such as case workers, a group suggested an idea to conduct a health economics study to quantify the need of such an intervention. Such a study may involve clinical and quality of life metrics, which may form the basis to influence policy renewal within this area.

(3) *Aesthetics:* Appearance was a consistent topic that was discussed by all groups, with one user sharing that “*appearance vs. function, it is a trade-off* ,” while another remarked: “*making it look more cosmetic seems to reduce the functionality*.” There were also discussions on whether prosthetic hands should look natural, by replicating skin tone. However, one user had a positive opinion about the unnatural appearance of prosthetic devices, as the visual appearance became an “interesting talking point in social situations.”

Opinions and experiences of aesthetics evolve over time, with one participant stating that “*aesthetics need to change with age*.” The topic of personalisation was discussed by two groups, with an emphasis on devices being unique to the user. For example, providing the option to customise and implement user personality into the visual appearance of a prosthesis, for instance by the design of the socket or the covering of the prosthetic hand.

Awareness of available aesthetic options and having a viewpoint as to what the prosthesis will look like before a user receives one, was noted by one group. This group stated that informing users of aesthetical properties before they receive a prosthesis may help to reduce an unknown aspect of the user experience. Also, understanding user needs about aesthetical parameters is a process that develops over time, between a user, their clinical team, designers, manufacturers and academics.

(4) *Clinical Service:* Clinical service was discussed in the context of the current care pathway in the UK and elsewhere, or in comparison to previous experiences. In particular, it was agreed that service users should be able to readily access information about all care options. Also, the requirement to inform close family and friends about the care and treatment options that are available and the experiences that may unfold for all involved was discussed. This was within the context of before, and after amputation. Furthermore, a specific point of discussion was about the varying types of pain that one may experience, such as phantom limb pain and chronic pain, and associated care options.

The expectations that people have of what the national healthcare provider can feasibly provide may often be “*unrealistic*,” which emphasises a need to inform users and the general public on the reality of living with limb difference or absence, through media coverage and awareness campaigns. Transparency of information between clinical teams and users was mentioned, especially when a prosthesis is being repaired: “*there is a severe lack of information provided as to where the arm is, or when I can expect to get it back*.”

The need to focus on the individual user and consider their personal experience and tailor care pathways accordingly was highlighted as a requirement going forward. This approach encompasses a broad scope of care, including physical and psychological rehabilitation, along with assessment strategies that determine what, when, and if a prosthesis is required.

*Opportunity:* Clinical service was identified as the most important theme by Group 3 at the end of Phase 1, to explore in Phase 2 (Figure 1). The opportunity focused on providing a clinical service that is specific, defined and consistent. This would potentially reduce the scenario of users receiving conflicting advice and opportunities.

(5) *Collaboration:* Collaboration is evolving to incorporate more sectors. However, not all types of collaboration are as common, as one industry specialist stated there are “very good collaborations between industry and the NHS [care provider], and between University and the NHS [care provider], but not so good between universities and industry.” Groups agreed that involving users in the collaboration process is beneficial in gaining real world perspective on living with limb difference or absence.

Factors limiting collaboration were identified, such as ethics. One workshop participant shared that “*ethics and processes stunt development and collaboration*.” One workshop participant voiced the opinion that clinicians should have the “*chance to do research*.”

None of the three users from one group were aware of the “*behind the scenes*” collaborations between clinicians and manufacturers. This reinforced the notion for transparency of information, which another group stated could be enabled by broader media coverage.

Collaboration was also discussed regarding creating peer networks, such as those used in other domains (e.g., exercise groups and psychological support amongst a trusted community). A consistent message across all groups was the need to adopt a more user-centred approach to projects, affirming the importance of forming strong collaborations.

*Opportunity:* Group 4 chose collaboration as the most important theme at the end of Phase 1, to explore in Phase 2 (Figure 1). The opportunity identified the potential of creating a user-centred treatment/care feedback loop, which encourages change, development, and innovation. A mechanism to enable this opportunity to be realised could be through the development of regional upper limb hubs that bring a variety of stakeholders together.

(6) *Media:* Media was discussed in both negative and positive terms by all groups. It was pointed out that media can both be a source of information, and an opportunity to relate information to the wider public. Groups talked about how media has informed a wider audience about prosthetic devices and future technology, which has led to broader public acceptance. Examples include the televised coverage of Paralympic Games. However, it was widely noted across all groups that media can create a misrepresentation of the reality of people living with limb loss or absence: “*you are not getting to hear the problems people face, or information on alternatives that may work better*.” This misrepresentation can lead to unrealistic expectations: “*the media is good for finding information on what is new, and you can get a lot of information online. But you do get friends sending you information on what the new bionics can do*.” These discussions led to four groups suggesting that friends and family members should be more involved in the care pathway that users receive, to build their knowledge and awareness of what is available and therefore what is currently possible.

There was an appetite to share or receive realistic information that represents the challenges of living with limb difference or absence. A group discussed an approach to address this opportunity, by creating a peer support community, so that people can share stories and ideas. Social media platforms could facilitate these networks, enabling users to connect and relate their own experience.

*Opportunity:* Group 2 chose media as the most important theme at the end of Phase 1, to explore in Phase 2 (Figure 2). The group described their collective decision of choosing media as a “*reluctant*” choice, relative to the other five themes. However, the group perceived media as a positive tool to influence and drive change for the other five themes, based on sharing information and authentic user stories more widely.

## Discussion

### Background

This paper presents findings from a user survey and a co-creation workshop that involved users, academics, clinicians, industry experts, and charity executives. The workshop, entitled: “The Future of Prosthetics: A User Perspective,” explored challenges and opportunities for the next ten years, with a focus on addressing user needs.

The last two decades have seen an increased academic interest in user needs and satisfaction (Postema et al., 1999, 2016; Davidson, 2002; Biddiss and Chau, 2007a,b; Biddiss et al., 2007; Kyberd et al., 2007; Jang et al., 2011; Østlie et al., 2012; Engdahl et al., 2015, 2017; Luchetti et al., 2015; Cordella et al., 2016; Chadwell et al., 2018; Sugawara et al., 2018; Kumar et al., 2019). However, solutions that address identified user needs do not seem to lead to a reduction in device abandonment (Kumar et al., 2019). Collaboration between academics and users, throughout the research process, can lead to the development of solutions that address user needs, therefore potentially leading to a reduction in device abandonment. An approach to facilitate this form of collaboration is co-creation, which facilitates the development of knowledge between academics and multiple stakeholders, such as users and clinicians (Cairney and Oliver, 2017; Hickey et al., 2018). The workshop presented here fits within the broad scope of co-creation (Pearce et al., 2020), as a multi-stakeholder collaborative event, which focused on identifying opportunities based on user perspectives and needs.

The workshop was organised into multi-stakeholder groups and focused on discussing the themes of function, psychology, aesthetics, clinical service, collaboration, and media. Despite the differences between the themes, common topics emerged from the group discussions, for example:

- Involving users in the research, development, and implementation phases of projects.
- Transparency of information between all stakeholders.
- Networks of support such as peer groups, in aid of psychological rehabilitation.
- Routes to informing healthcare policy through collaboration.

Based on the groups' discussions, function, clinical service, collaboration, and media were the top prioritised themes to explore further in Phase 2 (Figure 1). The groups navigated toward exploring the requirements to address challenges as multi-stakeholder teams, with users at the core of a teams' setup. This presents a situation where users become integral members of a research team (Brown, 2019). This notion was identified as an area for development over the next decade for the field of upper limb prosthetics, which may lead to an increase in translational research (Jones et al.).

### Co-creation With Multiple Stakeholders in Upper Limb Prosthetic Research

To enable research to translate into clinical practise, collaboration with policy makers may be beneficial, and lead to research impact (Maybin, 2015). However, the practicalities of academic research informing policy can be multi-faceted. The nature of academic funding, workflows, and research outputs may lead to a prolonged timescale for gathering evidence to inform policy. This is in contrast to the pace at which policy operates, which tends to be faster compared to academia (Maybin, 2015). Furthermore, experiential and practical knowledge as evidence is showing increased value within the policy domain (Oliver et al., 2019). Therefore, the collection of data on real world use of prostheses could inform policy and enhance clinician and user interaction. Gathering such evidence may require user data from an environment external to the laboratory, i.e., within the home (Simon et al., 2019).

As prosthetic research transitions toward a combination of laboratory-based studies and long-term trials in users' home environment; an opportunity to collaborate with users to gain input that contributes to the study can occur. For example, developing experimental protocols that take ethical considerations, such as privacy, into account whilst researching within user home environments (Jones et al.). In-home trials for upper limb prosthetics have been documented (Brinton et al., 2020; Chadwell et al., 2020; Garske et al., 2021; Wu et al., 2021), however there are challenges when conducting such studies with users. Acquiring ethical approval for user involvement within such studies, may present new scenarios for review committees to consider (Goodyear-Smith et al., 2015). For example, the iterative nuance of co-creation can lead to unpredictable outcomes, which may not fit with the pre-defined nature of ethical approval procedures. However, implementing on-going consent throughout a study provides an opportunity for users to periodically assess their involvement (Grant et al., 2019). Research teams should be aware of the long-term practicalities of implementing co-creation, and begin by navigating fundamental aspects, such as *power sharing* within multi-stakeholder teams (Hickey, 2018; Hickey et al., 2018), and how to establish joint decision making within studies (McKercher, 2020). Furthermore, acknowledging peoples' time, knowledge, and expertise beyond payment for travel and accommodation is a pertinent ethical consideration. For example, ensuring that payment rationale does not influence peoples' decision-making, and does not affect the risk and benefit of participating within a study (Tseng and Angelos, 2017). When face-to-face interaction is required, logistical and geographical barriers may occur (Brett et al., 2014; Domecq et al., 2014; Langley et al., 2018). In contrast, when online communication is required, accessibility to equipment, and technological knowledge becomes prevalent (Seah, 2020). However, researching with users, presents the potential to develop prosthetic devices that address user needs, through studies such as in-home trials, which in-turn can reduce the rate of abandonment within the field (Cordella et al., 2016).

In addition to the opportunity of collaborating with users, and policy makers; research teams can benefit from forming industrial partnerships, however there is reported loss of academic freedom (Rasmussen et al., 2018). An industry expert, who attended the workshop, shared the viewpoint that academic projects are not wholly conducive to forming a business plan for academic-industry partnerships. Furthermore, academic funding councils can steer researchers toward seeking answers to broad questions, which may not translate into industry or clinical practise in the short term.

The variety of focus of each aforementioned stakeholder group can present challenges when collaborating within academic research studies. Therefore, the requirement to implement an approach to facilitate such collaborations becomes prevalent. Co-creation can be an approach that enables researchers to collaborate with users, academics, clinicians, industry, charity, and policy makers. Conducting co-creation within research practise may require broad systemic development to support such approaches (Williams et al., 2020). The user survey and multi-stakeholder workshop that are presented within this paper, illustrates a starting point of a long-term vision of conducting co-creation throughout research studies in the field of upper limb prosthetics.

### Reflection

The pre-workshop survey presented inconsistency in how people responded to prioritising the themes. Therefore, the survey results may have yielded a different theme priority order, if all participants had completed the survey consistently. However, it is interesting to highlight that function was prioritised by two groups at the end of Phase 1B in the workshop.

The workshop model, based on the Double Diamond model (2019), was chosen based on the combination of divergent and convergent thinking. This enabled the workshop groups to explore the themes, and form conclusions within the time constraints of a 1-day workshop. This is one example of a workshop model for co-creation; other models exist within the literature, based on aspects such as joint decision making and collaboration (Jones, 2018).

In the preparation for the workshop, we invited participants from a range of relevant stakeholder groups in addition to users. Many other stakeholders could have been invited, for example, government representatives, policy makers, members of the advocacy groups or trade associations, and representatives from law firms and insurance companies. All of whom could offer valuable insight about care packages for prosthesis users. However, it would have been unrealistic to assume that the 1-day workshop could cover the range of themes with additional stakeholders. Future workshops may focus on fewer themes with additional stakeholder involvement. Furthermore, additional themes may emerge during future studies, which may be dependent upon the geographical region and associated care pathways for upper limb prosthetics. For example, potential themes could be costs and funding.

All workshop participants were based in UK. Therefore, some users' experiences, are influenced by how health and care is delivered in the UK. Nonetheless, we believe that many of the key learnings, e.g., the importance of prosthetic function and psychological factors, transcend beyond borders.

Finally, we acknowledge that the way prosthetic care and services are offered to war veterans is different to civilians. Hence their experiences may be different. This paper is centred upon experiences of care provided by the NHS, therefore war veterans were not involved in this particular study. However, it is likely that war veterans would also find the identified and discussed six themes pertinent.

## Conclusions

By gaining direct user perspectives, six themes were identified as relevant to their everyday experiences and needs. During a co-creation workshop, challenges and opportunities within these themes were explored by multiple stakeholders: users, academics, clinicians, industry experts, and charity executives.

The workshop highlighted function, collaboration, clinical service, and media as the most important themes for stakeholders to focus on over the next decade. To facilitate the translation of the workshop opportunities into a clinical setting, multi-stakeholder collaboration throughout the research process may be beneficial, namely between academics, users, clinicians, and policy makers. By implementing a co-creation approach, this form of collaboration could be realised, potentially leading to solutions that directly address user needs, with a subsequent decline in the rate of prosthesis device abandonment.

## Data Availability Statement

All data is included in this article. Further inquiries can be directed to the corresponding authors.

## Ethics Statement

This study was reviewed and approved by Faculty of Science, Agriculture & Engineering Research Ethics Committee, Newcastle University. Informed consent to participate in this study was provided by the participants.

## Author Contributions

HJ, SDu, and KN conceptualized this study, led the user survey and workshop, and visualized the figures and tables. HJ produced the original draft manuscript. All authors have contributed to the summary of the presented results and the discussion points and contributed to the review and editing of the manuscript.

## Conflict of Interest

The authors declare that the research was conducted in the absence of any commercial or financial relationships that could be construed as a potential conflict of interest.
